# COP1 Controls Abiotic Stress Responses by Modulating AtSIZ1 Function through Its E3 Ubiquitin Ligase Activity

**DOI:** 10.3389/fpls.2016.01182

**Published:** 2016-08-03

**Authors:** Joo Y. Kim, In-Cheol Jang, Hak S. Seo

**Affiliations:** ^1^Department of Plant Science, College of Agricultural Life Science, Seoul National University, SeoulSouth Korea; ^2^Temasek Life Sciences Laboratory, 1 Research Link, National University of Singapore, SingaporeSingapore

**Keywords:** abiotic stress, AtSIZ1, COP1, E3 ubiquitin ligase, E3 SUMO ligase, post-translational modification, SUMO, SUMO conjugate

## Abstract

Ubiquitination and sumoylation are essential post-translational modifications that regulate growth and development processes in plants, including control of hormone signaling mechanisms and responses to stress. This study showed that COP1 (Constitutive photomorphogenic 1) regulated the activity of *Arabidopsis* E3 SUMO (Small ubiquitin-related modifier) ligase AtSIZ1 through its E3 ubiquitin ligase activity. Yeast two hybrid analysis demonstrated that COP1 and AtSIZ1 directly interacted with one another, and subcellular localization assays indicated that COP1 and AtSIZ1 co-localized in nuclear bodies. Analysis of ubiquitination showed that AtSIZ1 was polyubiquitinated by COP1. The AtSIZ1 level was higher in *cop1-4* mutants than in wild-type seedlings under light or dark conditions, and overexpression of a dominant-negative (DN)-COP1 mutant led to a substantial increase in AtSIZ1 accumulation. In addition, under drought, cold, and high salt conditions, SUMO-conjugate levels were elevated in DN-COP1-overexpressing plants and *cop1-4* mutant plants compared to wild-type plants. Taken together, our results indicate that COP1 controls responses to abiotic stress by modulation of AtSIZ1 levels and activity.

## Introduction

As sessile organisms, plants are unable to control or avoid their surrounding environment. Plants therefore need to adapt to their environment by responding appropriately to stress and modulating their developmental programs. Many fine-tuning mechanisms are involved in adaptive changes, and, of these, the modification of translated proteins by the attachment of molecules such as ubiquitin, SUMO (Small ubiquitin-related modifier), and phosphate and methyl groups is thought to be a particularly rapid and efficient way to cope with environment changes.

Ubiquitination is a post-translational modification in which ubiquitin is covalently attached to a target protein by the activities of three enzymes: E1, E2, and E3 (Hochstrasser, 1996). Ubiquitination regulates several cellular processes such as cell division and growth, signal transduction, apoptosis, membrane trafficking, and DNA repair (Pickart, 2001; Swatek and Komander, 2016). Two different ubiquitination pathways have been reported to date. In the first pathway, monoubiquitination, a single ubiquitin molecule is attached to a target protein. In the second pathway, polyubiquitination, target proteins are conjugated by polyubiquitins. Usually, monoubiquitinated proteins regulate important cellular functions such as histone processes, endocytosis, and virus budding (Hicke, 2001; Swatek and Komander, 2016), whereas polyubiquitinated proteins are mainly targeted for degradation by the 26S proteasome complex (Pickart, 2001; Haglund and Dikic, 2005; Swatek and Komander, 2016). Ubiquitin contains seven lysine (K) residues, K6, K11, K27, K29, K33, K48, and K63, and all of them can form an isopeptide linkage with a target protein (Komander, 2009). Ubiquitin chains can be arranged in different styles, which results in specific outcomes for the specific substrate. For example, monoubiquitination and K63 polyubiquitination are involved in regulating protein activation or signal transduction, while K6 and K48 polyubiquitination targets proteins for proteasomal degradation (Ikeda and Dikic, 2008).

Sumoylation, another important post-translational modification, involves covalent conjugation of a small modifier, SUMO, to target lysine residues. As with ubiquitination, sumoylation occurs as a result of the activities of three enzymes: E1, E2, and E3 (Gill, 2004; Kerscher et al., 2006; Geiss-Friedlander and Melchior, 2007). In most cases, sumoylated proteins are not degraded. Sumoylation of target proteins affects their subcellular localization, function, and stability, and also influences their involvement in cellular processes such as mitochondrial dynamics and the response to DNA damage (Livnat-Levanon and Glickman, 2011; Cubeñas-Potts and Matunis, 2013; Yates et al., 2016).

SIZ1, an E3 SUMO ligase, contains a RING-like domain, Siz-PIAS RING (SP-RING), that interacts with SUMO-conjugating E2 enzyme and a chromatin organization domain termed SAF-A/B-Acinus-PIAS (SAP; Jackson, 2001). *Arabidopsis* E3 SUMO ligase AtSIZ1 regulates growth and development and has roles in nutrient assimilation, hormone signaling, and flowering (Miura et al., 2005, 2010; Jin et al., 2008; Park et al., 2011; Son et al., 2014; Kim D.Y. et al., 2015; Kim S.-I. et al., 2015; Kim et al., 2016). AtSIZ1 also affects responses to abiotic stresses in plants. For example, AtSIZ1 knock-out mutants exhibited increased susceptibility to low temperature, drought, heat, and salt stresses (Yoo et al., 2006; Catala et al., 2007; Miura et al., 2007, 2011), and AtSIZ1-overexpressing transgenic plants exhibited tolerance to cold and salt stresses (Miura and Nozawa, 2014). Moreover, creeping bentgrass overexpressing rice E3 SUMO ligase OsSIZ1 was resistant to drought and heat stresses (Li et al., 2013). These results suggest that AtSIZ1 has crucial functions in plant adaptations to stress.

COP1 (Constitutive photomorphogenic 1), an E3 ubiquitin ligase, contains RING-finger, coiled-coil, and WD40 domains (Deng et al., 1992) and participates in signal transduction and responses to stress via regulation of the stability of various proteins in plant and animal cells (Yi and Deng, 2005). In plants, COP1 ubiquitinates photomorphogenic promoting factors, which leads to their degradation and downstream repression of photomorphogenesis. Previous research identified several COP1 substrates in plants. Activity levels of HY5 (Long hypocotyl 5), HFR1 (Long hypocotyl in far-red 1), LAF1 (Long hypocotyl after far-red light 1), PHYA (Phytochrome A), PHYB (Phytochrome B), CRY1 (Cryptochrome 1), CRY2 (Cryptochrome 2), PIL1 (Phytochrome interacting factor 3-like 1), CO (CONSTANS), GI (GIGANTEA), and ELF3 (Early flowering 3) were modulated by the E3 ubiquitin ligase activity of COP1 (Osterlund et al., 2000; Wang et al., 2001; Yang et al., 2001; Seo et al., 2003, 2004; Jang et al., 2005, 2008, 2010; Yu et al., 2008; Luo et al., 2014). These modulated activities suggested a role for COP1 in the control of seedling development, flowering time, and circadian rhythms. In addition, COP1 was found to be involved in plant defenses against virus attack, root development, hormone signaling, and miRNA biogenesis (Jeong et al., 2010; Luo et al., 2010; Dyachok et al., 2011; Chico et al., 2014; Cho et al., 2014).

Recent studies suggested that the sumoylation system was associated with the ubiquitination system. For example, sumoylation of mouse double minute 2 homolog (Mdm2) prevented its ubiquitination (Buschmann et al., 2000). Separate research showed that some polysumoylated proteins were ubiquitinated by SUMO-targeted ubiquitin ligases (STUbLs; Sriramachandran and Dohmen, 2014), demonstrating that the SUMO chain could act as a recognition signal for E3 ubiquitin ligases. Slx5/Slx8, a type of STUbL, directly interacted with E3 SUMO ligase and thereby mediated protein degradation by the proteasome complex (Westerbeck et al., 2014). These data suggest that there are unidentified regulatory pathways for E3 SUMO ligase and E3 ubiquitin ligase remaining to be discovered, and suggest that AtSIZ1 and COP1 may regulate the functions of each another. To address this question, the ability of COP1 to control AtSIZ1 levels and activity was examined in this study.

Our data indicate that COP1 has E3 ubiquitin ligase activity for AtSIZ1. Down-regulation of COP1 activity leads to AtSIZ1 accumulation, which induces SUMO conjugation of target proteins under abiotic stress conditions.

## Materials and Methods

### Plant Growth Conditions and Stress Treatments

*Arabidopsis thaliana* ecotype Col-0, *cop1-4*, and dominant-negative (DN)-COP1 transgenic plant tissues were obtained from plants grown on solid MS medium at 22°C under a 16 h light/8 h dark lighting regime. For stress treatments, plants were grown as above, then transferred to liquid media for 2 days to allow adaptation. To initiate temperature stress, cultures were transferred to shaking water baths at 4°C for the indicated time. For drought stress, plants grown on MS were directly exposed to air at 22°C under continuous light. For salt stress, 200 mM NaCl was added directly to the liquid culture medium.

### Yeast Two Hybrid Experiments and Preparation of Recombinant Proteins

Sequences encoding full-length COP1, deletion mutants (WD40, amino acids 1–255; RING/Coiled-coil, amino acids 110–675; RING/Coiled-coil, amino acids 216–675), full-length AtSIZ1, full-length E2 SUMO-conjugating enzyme AtSCE1a, and full-length SUMO1 were amplified by PCR and cloned into pGBT8 or pGAD424 (New England Biolabs) to generate cDNAs encoding proteins fused with GAL4 DNA-binding or -activating domains. Yeast two hybrid experiments were performed as previously described (Xie et al., 2002). Sequences for a C-terminal HA tag were appended to the full-length AtSIZ1 cDNA by PCR and cloned into pRSET A (Invitrogen) to generate the coding sequence for His_6_-AtSIZ1-HA. Proteins were expressed in *Escherichia coli* strain BL21 and purified as previously described (Seo et al., 2003). cDNA encoding full-length COP1 fused with maltose-binding protein (MBP) were prepared as previously described (Seo et al., 2003).

### Antibody Production and Western Analysis

Polyclonal anti-AtSIZ1 antiserum was obtained from rabbit immunized with His_6_-tagged C-terminal AtSIZ1 (amino acids, 421–873) expressed and purified from *E. coli*. Anti-AtSIZ1 antibodies were affinity-purified from serum by adsorption to AtSIZ1 protein bound to nitrocellulose membrane. Western blots were performed according to the manufacturer’s instructions (Amersham Bioscience).

### *In vitro* Ubiquitination Assays

*In vitro* ubiquitination was performed using 100 ng of purified His_6_-AtSIZ1-HA, as previously described (Seo et al., 2003). After incubation at 30°C for 2 h, reaction mixtures were separated on 6% SDS-PAGE gels. Ubiquitinated His_6_-AtSIZ1-HA and MBP-COP1 were detected by Western blot analysis using anti-HA antibody or anti-MBP antibody (Santa Cruz Biotechnology).

### Effects of COP1 Overexpression on AtSIZ1 Levels *In vivo*

Seven-days-old plants carrying *XVE-DN-COP1-Myc_6_* transgenes (Seo et al., 2004) grown on MS media were treated in the light with or without 10 μM β-estradiol for 15 h. Samples were ground in liquid nitrogen, and equivalent amounts were analyzed by Western blot analysis using anti-AtSIZ1 antibody or anti-Myc antibody (Santa Cruz Biotechnology).

### Examination of the AtSIZ1 Level in *cop1-4* Mutants

Four-days-old wild-type and *cop1-4* mutant seedlings grown under light or dark conditions on MS media were ground in liquid nitrogen, and equivalent amounts were analyzed by Western blot analysis using an anti-AtSIZ1 antibody.

### Detection of SUMO Conjugates

Cold-, drought-, and salt-stress-treated samples were ground in liquid nitrogen, and equivalent amounts were analyzed by Western blot analysis using anti-AtSUMO1 (Abcam).

## Results

### COP1 and AtSIZ1 Co-localize in Nuclear Bodies

**FIGURE 1:**
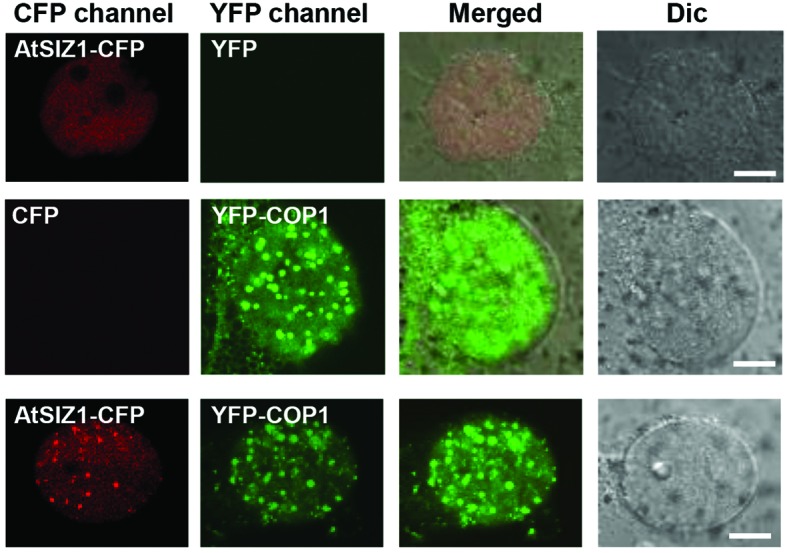
**AtSIZ1 and COP1 co-localize in nuclear bodies.** AtSIZ1-CFP and YFP-COP1 were transiently expressed in onion epidermal cells. YFP-COP1 and AtSIZ1-CFP were distributed in the nucleus when expressed independently. Simultaneously expressed proteins were co-localized in nuclear bodies.

AtSIZ1 was previously shown to localize in the nucleus (Miura et al., 2005). COP1 was also found in the nucleus, within nuclear bodies (von Arnim and Deng, 1994; Seo et al., 2003, 2004), but moved to the cytoplasm when exposed to light (Stacey et al., 1999). The similar locations of the two proteins suggested that interactions could occur between COP1 and AtSIZ1 in the nucleus. We therefore examined the cellular localization patterns of COP1 and AtSIZ1 to determine whether the two proteins co-localized in plant cells. YFP-COP1 and AtSIZ1-CFP were transiently expressed in onion epidermal cells. As expected, YFP-COP1 and AtSIZ1-CFP were distributed in the nucleus when expressed individually (**Figure 1**). Co-expression of YFP-COP1 and AtSIZ1-CFP revealed that the two proteins localized in the same nuclear bodies; however, the AtSIZ1-CFP signal was relatively weak within nuclear bodies and the majority of AtSIZ1-CFP was distributed throughout the nucleus (**Figure 1**). We thus counted the number of speckles in the AtSIZ1-CFP and YFP-COP1 panels. In total, 29 and 42 speckles were clearly detected in the AtSIZ1-CFP and YFP-COP1 panels, respectively, indicating that the number of speckles that colocalized in both the AtSIZ1-CFP and YFP-COP1 panels was 29. This means that approximately 69% of YFP-COP1 speckles colocalized with AtSIZ1-CFP speckles.

### AtSIZ1 Physically Interacts with COP1

**FIGURE 2:**
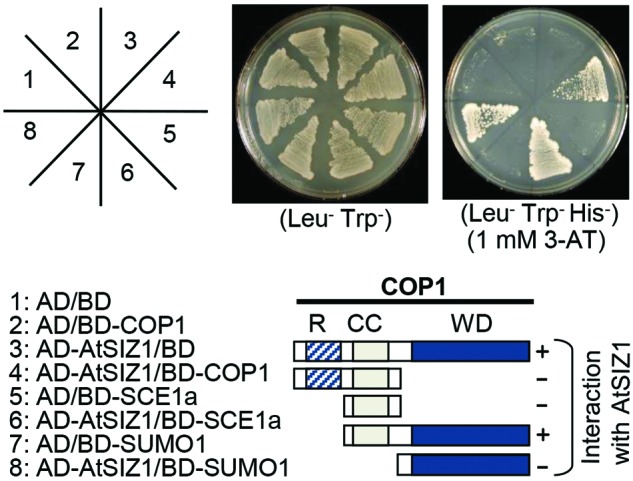
**AtSIZ1 and COP1 directly interact *in vivo*.** AtSIZ1 cDNA and several different COP1 cDNA fragments were fused to sequences encoding the Gal4 activation domain (AD) or the Gal4 DNA-binding domain (BD) in pGAD424 and pGBT8, respectively. COP1 mutant proteins are indicated on the schematic diagrams. AtSIZ1 and COP1 constructs were co-transformed into yeast strain AH109. Transformants were plated onto minimal medium Leu/-Trp or Leu/-Trp/-His, including 1 mM 3-AT, and incubated for 4 days. R, CC, and WD refer to RING motif, coiled-coil domain, and WD40 domain, respectively.

Co-localization of COP1 and AtSIZ1 in nuclear bodies suggested that the two proteins might be able to interact with one another. Yeast two hybrid analysis was used to test whether such interactions could occur. The WD40 domain of COP1 interacted strongly with AtSIZ1 (**Figure 2**). However, when COP1 was modified to remove the coiled-coil domain responsible for dimerization, no interaction with AtSIZ1 was observed (**Figure 2**), indicating that both WD40 and dimerization domains are required for interaction with AtSIZ1. AtSIZ1 strongly interacted with both SAE1 and SUMO1, which were used as positive controls (**Figure 2**).

### AtSIZ1 Is Ubiquitinated by COP1 *In vitro*

**FIGURE 3:**
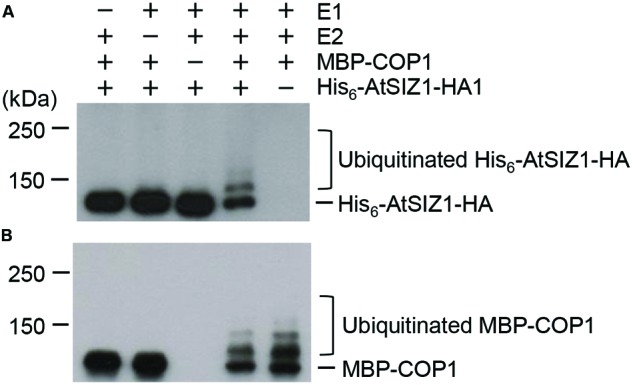
**AtSIZ1 is ubiquitinated by COP1 *in vitro*.** Ubiquitination reactions were performed in the presence or absence of rabbit E1, UbcH5b (E2), MBP-COP1 (E3), His_6_-Ubiquitin, and His_6_-AtSIZ1-HA. After reactions were complete, ubiquitinated AtSIZ1 **(A)** and COP1 **(B)** were detected by Western blot analysis using anti-HA and anti-MBP antibodies, respectively.

The direct interaction of AtSIZ1 and COP1 and their co-localization provided preliminary evidence that AtSIZ1 could be a substrate for COP1. To investigate this further, recombinant His_6_-AtSIZ1-HA and MBP-COP1 proteins were expressed in *E. coli*, purified using nickel or amylose columns, and used for *in vitro* ubiquitination reactions. AtSIZ1 was polyubiquitinated by COP1 (**Figure 3A**), indicating that COP1 had E3 ubiquitin ligase activity for AtSIZ1. Polyubiquitinated COP1 was also detected (**Figure 3B**), proving that COP1 was active during the reaction.

### AtSIZ1 Levels Are Regulated by COP1 *Invivo*

**FIGURE 4:**
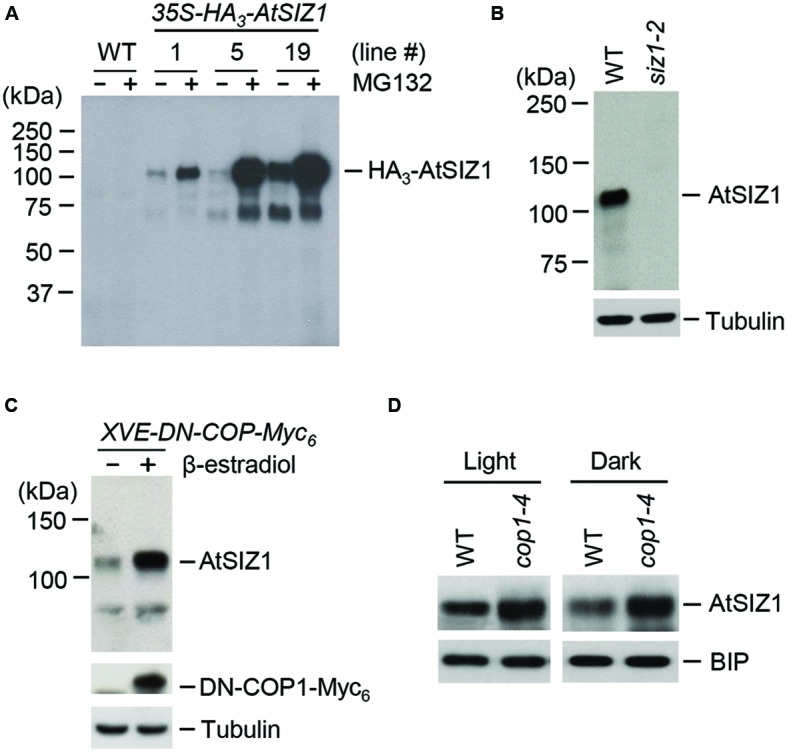
**AtSIZ1 is unstable and negatively regulated by COP1. (A)** Degradation of AtSIZ1 by the proteasome pathway. Transgenic plants transformed with *35S-AtSIZ1-HA3* were treated with MG132, and AtSIZ1 levels were examined by Western blot analysis using an anti-HA antibody. Three different transgenic lines were used for the experiment. **(B)** Specificity of the anti-AtSIZ1 antibody. Total proteins were extracted from 2-weeks-old wild-type and *siz1-2* mutant plants, and AtSIZ1 was detected by Western blot analysis using the anti-AtSIZ1 antibody. Tubulin was used as a loading control. **(C)** Effect of COP1 overexpression on AtSIZ1 levels. Two-weeks-old *XVE-DN-COP1-Myc_6_* transgenic plants were treated with β-estradiol, and AtSIZ1 levels were determined by Western blot analysis using an anti-AtSIZ1 antibody. Tubulin was used as a loading control. **(D)** Effect of COP1 on AtSIZ1 levels *in vivo*. Wild-type seedlings and *cop1-4* mutants were grown for 4 days under light or dark conditions, and AtSIZ1 levels were determined by Western blot analysis using an anti-AtSIZ1 antibody. BIP (binding protein) was used as a loading control.

The ubiquitination of AtSIZ1 *in vitro* suggested that AtSIZ1 could be polyubiquitinated by COP1 in plants and, as a consequence, be degraded by the 26S proteasome complex. We therefore asked if AtSIZ1 could be degraded by the 26S proteasome complex. Transgenic plants were generated in which AtSIZ1 was overexpressed under the control of the 35S promoter. These plants were then treated with a 26S proteasome inhibitor, MG-132. AtSIZ1 levels increased after MG-132 treatment, indicating that AtSIZ1 was degraded by the 26S proteasome complex after polyubiquitination (**Figure 4A**). An anti-AtSIZ1 antibody was produced for use in experiments to determine whether COP1 had a direct effect on AtSIZ1 degradation. The specificity of the antibody was tested by Western blot analysis of wild-type and *siz1-2* mutant plants. AtSIZ1 was detected in wild-type plants but not in the *siz1-2* mutants, indicating that the anti-AtSIZ1 antibody specifically recognized AtSIZ1. The effect of COP1 on AtSIZ1 levels was determined. Transgenic plants were produced that carried a β-estradiol-inducible DN-COP1 transgene that was mutated in the RING-finger motif (Seo et al., 2004). AtSIZ1 levels increased after induction of DN-COP1 by β-estradiol, as determined by Western blot analysis (**Figure 4C**). We also examined the AtSIZ1 level in wild-type and *cop1-4* mutant seedlings, which were grown for 4 days under light or dark conditions. As expected, the AtSIZ1 level was higher in *cop1-4* mutants than in wild-type seedlings under both conditions (**Figure 4D**), indicating that COP1 negatively regulated AtSIZ1 function.

### COP1 Controls SUMO Conjugation under Various Stress Conditions

**FIGURE 5:**
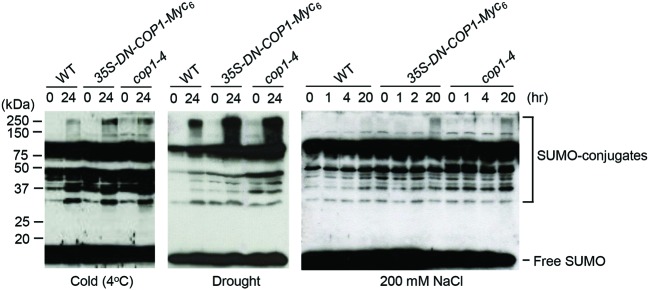
**Accumulation of SUMO conjugates in DN-COP1-overexpressing plants and *cop1-4* mutant plants under abiotic stress conditions.** Wild-type plants, DN-COP1-overexpressing plants, and *cop1-4* mutant plants were exposed to cold stress (4°C), drought stress, and salt stress (200 mM NaCl) for the indicated times. SUMO conjugates were detected by Western blot analysis with anti-AtSUMO1 antibody.

To identify the effect of COP1 on sumoylation under stress conditions, SUMO-conjugate levels were examined in wild-type, *cop1-4* mutant, and DN-COP1-overexpressing plants exposed to cold (4°C), drought, and high salt (200 mM NaCl) stresses. Previous research reported accumulation of SUMO conjugates in *Arabidopsis* under similar conditions (Kurepa et al., 2003). As expected, SUMO-conjugate levels were elevated in DN-COP1-overexpressing plants and *cop1-4* mutant plants compared to wild-type plants (**Figure 5**). This indicated that the accumulation of SUMO conjugates in DN-COP1-overexpressing plants and *cop1-4* mutants resulted from AtSIZ1 stabilization, which occurred as a result of loss of COP1 activity.

## Discussion

Cellular ubiquitination systems regulate various cellular events either by prompting protein destabilization or by changing the activity and function of modified proteins (Swatek and Komander, 2016; Yates et al., 2016). This study showed that COP1 modulated AtSIZ1 levels via its E3 ubiquitin ligase activity and, in turn, modulation of AtSIZ1 levels affected the sumoylation of target proteins involved in the response to abiotic stresses.

COP1 mutants exhibit an aberrant photomorphogenic phenotype under dark growth conditions. Previous research focused on characterizing the functions of COP1 in light signaling, and several transcription factors and photoreceptors were shown to be negatively regulated by COP1 (Lau and Deng, 2012). Previous work also suggested that COP1 destabilized its interaction partners, but *in vitro* ubiquitination assays revealed that not all of the putative interaction partners were real COP1 substrates (Seo et al., 2003, 2004; Jang et al., 2005; Liu et al., 2008).

Unlike some ubiquitinylated proteins, proteins modified with SUMO are not degraded but instead take on important cellular functions (Callis, 2014; Yates et al., 2016). Several recent studies showed that E3 SUMO ligase activity was regulated by ubiquitination. For example, Slx5/Slx8, a STUbL, directly ubiquitinated E3 SUMO ligase SIZ1 in fission yeast and led to degradation of SIZ1 by the 26S proteasome complex (Westerbeck et al., 2014). STUbLs also recognized polysumoylated target proteins, thereby linking sumoylation to ubiquitinylation-mediated degradation (Sriramachandran and Dohmen, 2014). Similar results were recently reported in plants. For example, *Arabidopsis* STUbLs complemented the growth defects of the *Schizosaccharomyces pombe* STUbL mutant *rfp1*/*rfp2* to varying degrees (Elrouby et al., 2013; Yates et al., 2016). These findings strongly suggest that a range of E3 ubiquitin ligases can exhibit ligase activities for various E3 SUMO ligases. Subcellular localization showed that COP1 and AtSIZ1 localized in the nucleus (Seo et al., 2003, 2004; Son et al., 2014). On the basis of these studies, we reasoned that COP1 might be able to control AtSIZ1 levels and/or activity. Results in the current study showed that AtSIZ1 was polyubiquitinated by COP1 through direct interaction of the proteins (**Figure 3**), indicating that COP1 functioned as an E3 ubiquitin ligase for AtSIZ1. In addition, AtSIZ1 levels increased substantially when COP1 activity was repressed, indicating that AtSIZ1 levels were tightly controlled by COP1 activity (**Figure 4**). This is the first evidence in plants that an E3 SUMO ligase is modulated by the activity of an E3 ubiquitin ligase.

COP1 can directly ubiquitinate target substrates including transcription factors and phytochromes (Henriques et al., 2009; Jang et al., 2010). COP1 also forms a complex with the SUPPRESSOR OF PHYA-105 (SPA) protein, which enhances COP1 activity (Zhu et al., 2008). For example, COP1-SPA complexes directly ubiquitinate transcription factors such as HY5, LAF1, and HFR1 (Saijo et al., 2003; Seo et al., 2003; Yang et al., 2005). However, phytochrome-interacting factors (PIFs) are not directly ubiquitinated by COP1 (Jang et al., 2010; Xu et al., 2014). Recently, it was shown that PIF1 is ubiquitinated by the CUL4^COP1-SPA^ E3 ubiquitin ligase, a Cullin4 (CUL4)-COP1-SPA complex (Zhu et al., 2015), and the CUL4^COP1-SPA^ E3 ubiquitin ligase is also necessary for co-degradation of PIF3 and phyB (Ni et al., 2014). Moreover, PIF1 interacts with COP1-SPA1 E3 ubiquitin ligase complexes and increases E3 ligase activity for HY5 degradation (Xu et al., 2014). All of these data indicate that COP1 can directly or indirectly ubiquitinate target proteins alone or as a complex, and that COP1 activity can be regulated by its interacting factors. The data also indicate that COP1 can function as a cofactor of E3 ubiquitin ligase complexes including CUL4^COP1-SPA^. In our current study, the effect of COP1 on the AtSIZ1 level was evaluated without consideration of COP1-interacting factors including SPA1, CUL4, PIF1, and other proteins. Thus, further biochemical and genetic characterizations using COP1-interacting factors and their mutants will be required to elucidate the effect of COP1 activity on AtSIZ1 stability and function.

Light induces redistribution of COP1 or the COP1-SPA complex from the nucleus to the cytosol, which indicates that COP1 activity is light-dependent (Subramanian et al., 2004; Pacín et al., 2014; Sheerin et al., 2015). Recently, it was also shown that the CUL4^COP1-SPA^ E3 ubiquitin ligase is required for the light-induced degradation of PIF1 (Zhu et al., 2015). Our assays (**Figures 4A–D**) were performed using light- or dark-grown plants. Therefore, further experiments will be also needed to examine the effect of COP1 activity on the level and stability of AtSIZ1 using seedlings and mature plants after dark treatment of light-grown plants or light treatment of dark-grown plants.

Previous studies suggested that COP1 was involved in the response to abiotic stresses such as salt (Yu et al., 2016), cold (Jung et al., 2012), and UV-B exposure (Yin et al., 2015) and in the response to biotic stresses such as viral attack (Jeong et al., 2010; Chico et al., 2014). Sumoylation was also linked to the response to abiotic stresses such as cold, heat, and salt exposure (Yoo et al., 2006; Catala et al., 2007; Miura et al., 2007; Yates et al., 2016). Although some SUMO-conjugated proteins are desumoylated under stress conditions, a large number of proteins are modified by SUMO during the stress response (Miller et al., 2010; Yates et al., 2016). Our data showed that AtSIZ1 levels increased upon down-regulation or loss of COP1 activity (**Figures 4C,D**), suggesting that COP1 can control abiotic stress responses via the regulation of AtSIZ1-mediated sumoylation of target proteins.

Under low temperature, drought, and high salt conditions, SUMO-conjugate levels were higher in DN-COP1-overexpressing plants and *cop1-4* mutant plants compared to the wild type (**Figure 5**). These results strongly suggest that AtSIZ1-mediated sumoylation of target substrates is tightly controlled by COP1 activity.

## Conclusion

Our results showed that COP1 negatively regulated AtSIZ1 levels through its E3 ubiquitin ligase activity, thereby negatively regulating the AtSIZ1-mediated SUMO conjugation of target proteins under abiotic stress conditions. COP1 therefore modulated the abiotic stress responses controlled by the sumoylation system. In addition, the direct interaction between COP1 and AtSIZ1 suggested that COP1 levels and activity could be controlled by sumoylation via the E3 SUMO ligase activity of AtSIZ1. Further research concerning COP1-mediated AtSIZ1 function and AtSIZ1-mediated COP1 function will enhance our understanding of the mechanisms underlying the cross-control of the ubiquitination and sumoylation systems, and their involvement in the modulation of signal transduction and stress responses.

## Author Contributions

HS designed the project. JK, I-CJ, and HS carried out experiments. JK and HS analyzed and interpreted the data. JK and HS wrote the manuscript. All authors commented on the results and the manuscript.

## Conflict of Interest Statement

The authors declare that the research was conducted in the absence of any commercial or financial relationships that could be construed as a potential conflict of interest.
